# Monomeric C-Reactive Protein Localized in the Cerebral Tissue of Damaged Vascular Brain Regions Is Associated With Neuro-Inflammation and Neurodegeneration-An Immunohistochemical Study

**DOI:** 10.3389/fimmu.2021.644213

**Published:** 2021-03-16

**Authors:** Raid S. Al-Baradie, Shuang Pu, Donghui Liu, Yasmin Zeinolabediny, Glenn Ferris, Coral Sanfeli, Ruben Corpas, Elisa Garcia-Lara, Suliman A. Alsagaby, Bader M. Alshehri, Ahmed M. Abdel-hadi, Fuzail Ahmad, Psalm Moatari, Nima Heidari, Mark Slevin

**Affiliations:** ^1^Department of Medical Laboratory Sciences, College of Applied Medical Sciences, Majmaah University, Al Majma'ah, Saudi Arabia; ^2^National Natural Science foundation of China, Beijing, China; ^3^School of Healthcare Science, John Dalton Building, Manchester Metropolitan University, Manchester, United Kingdom; ^4^Instituto De Investigaciones Biomedicas De Barcelona, CSIC, Barcelona, Spain; ^5^Department of Physical Therapy and Health Rehabilitation, College of Applied Medical Sciences, Majmaah University, Al Majma'ah, Saudi Arabia; ^6^Salford Royal NHS Foundation Trust, Manchester, United Kingdom; ^7^The Regenerative Clinic, London, United Kingdom; ^8^University of Medicine, Pharmacy, Science and Technology, Târgu Mures, Romania

**Keywords:** vascular, neurodegeneration, C-reactive protein, stroke, inflammation

## Abstract

Monomeric C-reactive protein (mCRP) is now accepted as having a key role in modulating inflammation and in particular, has been strongly associated with atherosclerotic arterial plaque progression and instability and neuroinflammation after stroke where a build-up of the mCRP protein within the brain parenchyma appears to be connected to vascular damage, neurodegenerative pathophysiology and possibly Alzheimer's Disease (AD) and dementia. Here, using immunohistochemical analysis, we wanted to confirm mCRP localization and overall distribution within a cohort of AD patients showing evidence of previous infarction and then focus on its co-localization with inflammatory active regions in order to provide further evidence of its functional and direct impact. We showed that mCRP was particularly seen in large amounts within brain vessels of all sizes and that the immediate micro-environment surrounding these had become laden with mCRP positive cells and extra cellular matrix. This suggested possible leakage and transport into the local tissue. The mCRP-positive regions were almost always associated with neurodegenerative, damaged tissue as hallmarked by co-positivity with pTau and β-amyloid staining. Where this occurred, cells with the morphology of neurons, macrophages and glia, as well as smaller microvessels became mCRP-positive in regions staining for the inflammatory markers CD68 (macrophage), interleukin-1 beta (IL-1β) and nuclear factor kappa B (NFκB), showing evidence of a perpetuation of inflammation. Positive staining for mCRP was seen even in distant hypothalamic regions. In conclusion, brain injury or inflammatory neurodegenerative processes are strongly associated with mCRP localization within the tissue and given our knowledge of its biological properties, it is likely that this protein plays a direct role in promoting tissue damage and supporting progression of AD after injury.

## Introduction

Alzheimer's disease (AD) is the most common form of dementia, a brain degenerative disease affecting 1:14 of the population over 65 years of age (1). The disease is irreversible and current therapeutics can only slow down progression of the disease (2).

Our previous work showed that monomeric C-reactive protein (mCRP) is found in large amounts in the parenchyma following ischemic or hemorrhagic stroke and appears to originate mainly from blood vessel leakage into the local surrounding tissue (3, 4). Dementia of vascular origin is associated with damage to the deep penetrating arteries within the parenchyma and this can result from head trauma, ischemic, or hemorrhagic stroke or linked to genetic abnormalities particularly in younger patients (5, 6).

Chronic neuroinflammation is a key determinant of ongoing and future likelihood of development of cognitive impairment and subsequent risk of AD (7), whilst, acute vascular events associated with stroke, other injury or repeated minor disturbances found in lacunar stroke, also predispose individuals significantly to neurodegenerative complications (8, 9).

C-reactive protein in general, has been used particularly in the fields of cardiovascular disease and autoimmune conditions as well as sepsis to indicate levels of inflammation, however over the last decade, its monomeric form (mCRP) has been highlighted as the major biologically active form, and both circulating levels of CRP as well as tissue-associated mCRP within the brain have been shown to be associated with development of dementia (10).

mCRP strongly activates angiogenesis both *in vitro* and *in vivo* through a mechanism involving MAP kinase signaling and Notch-3, but this vasculogenic process appears to be flawed often producing in patent or leaky blood vessels (11). It is interesting therefore to examine the brain vasculature in relation to local inflammatory status as this may provide an insight into the developing neurodegenerative process.

Prior studies have demonstrated mCRP localization in the plaques of individuals with AD (12). Here, we investigated the link between mCRP and its co-localization within the brain and indicators of previous stroke or vascular disruption and dementia. In addition, we used co-labeling to confirm a direct association of mCRP-positive regions with neuro-inflammatory processes.

## Methods

### Tissue Sample Collection

Alzheimer's tissue obtained at post-mortem was obtained from a total of 13 patients (identified from a larger cohort by their co-existence of previous infarction as determined by histological pre-examination) where all hemispheres/regions of tissue were available from the Brain Bank in Bristol (UK). Tissue sections were cut from paraffin blocks of separated regions from the cerebral cortex, as follows: (1) frontal lobe, (2) parietal lobe, and (3) occipital lobe (Table 1). All cases had a history of progressive dementia some with evidence of stroke and each was selected on the basis of a diagnosis according to CERAD of “definite AD and a Braak tangle stage of V–VI; according to NIA-Alzheimer's Association guidelines, where the AD neuropathological change was the gold standard registering the patient as dementured.

**Table 1:** Table of patients/samples.

**Number**	**Age**	**Sex**	**Diagnosis-1**	**Diagnosis-2**
893F	71	M	AD	CAA
883BG	91	F	CVD	CONTROL-
916OCC	72	F	VaD	MELANOMA
850F	79	M	AD	CVD
798PAR	95	F	AD	CVD
954OCC	84	F	AD	CVD
839F	71	M	AD	CAA
963PAR	71	M	AD	CAA
855BG	93	F	TBI	CONTROL
815F	70	M	AD	–
697PAR	75	M	AD	CAA
839HYPO	81	M	AD	CAA
691F	77	F	VaD	–

*F, frontal; BG, basal ganglia; OCC, occipital; PAR, parietal; AD, Alzheimer's disease; CVD, cardiovascular disease; VaD, vascular dementia; CAA, cerebral amyloid angiopathy; TBI, traumatic brain injury; HYPO, hypothalamus. All samples were chosen from the brain bank on the basis that AD/VAD was the primary diagnosis at post-mortem. The selection here apart from the controls represent those that subsequent histology revealed an additional presence of infarct*.

Ethical approval was gained through application to https://mrc.ukri.org/research/facilities-and-resources-for-researchers/brain-banks/, and following their guidelines at https://mrc.ukri.org/publications/browse/human-tissue-and-biological-samples-for-use-in-research/; local approval was gained for the use of the tissue within the Manchester Metropolitan University LREC committee)-more details are shown in Slevin et al. (13), Mirra et al. (14), Braak et al. (15), and Montine et al. (16). Some of the patients had evidence of infarction from previous stroke, peri-infarcted tissue showed structural integrity that was characterized by oedema, altered morphology of the neurons (some showing changes of apoptosis), inflammatory macrophage infiltration and angiogenesis. Tissue with regular looking morphology served as a control.

### Stereotactic Hippocampal Injection of Mcrp in Mice and Histological Analysis

Methodological details for this previously conducted work, and treatment are provided in the article by Slevin et al. (13) where in Figure 4E of this already published article, an image is displayed showing mCRP-positive neurons in the hypothalamus of mice (shown here as **Figure 2E**). Further images from these experiments are provided in the results section to qualify this finding.

### Immunohistochemistry and Double Labeling Procedures

mCRP-specific monoclonal antibody 8C10 was prepared and supplied by Dr. Lawrence Potempa and fully characterized as shown in the article by Schwedler et al. (17).

Single and double immunofluorescence was performed after antigen retrieval (slides incubated for 40 min in 0.01 M of sodium citrate buffer at a pH of 6.0 and a temperature of 95°C), cooled at room temperature and stored for 30 min in a 2% hydrogen peroxide solution.

After blocking for 1 h in 10% goat serum, the slides were incubated alone or sequentially over night with anti- mouse monoclonal mCRP-8C10 antibodies (obtained as a gift from Professor Lawrence Potempa; 1:10 in 1% Goat serum/0.1% Tween 20/1x PBS) and then after washing, with either rabbit polyclonal CD68, IL-1β or phospho-NF-κB (InVitrogen, UK; 1:50 in 1% Goat serum/0.1% Tween 20/1x PBS) for 18 h at 4°C, followed after washings by the second antibody, detected using a mix of goat anti rabbit CFL 488 and goat anti-mouse CFL 568 (1:100, Santa Cruz, Heidelberg, Germany) in 1% Goat serum/0.1% Tween 20/1x PBS for 4 h at room temperature. Several color combinations were used in co-labeled sections depending on the need and these are described in the figure legends. Regions of tissue with normal looking morphology that in addition did not show mCRP staining from the same sections are included as controls.

Sections were mounted in DPX (Life Technologies, Karlsruhe, Germany) and slides were visualized on confocal microscope. Control cases were stained with a standard diaminobenzidine staining. Negative controls were included where the primary antibody was replaced with PBS- the primary antibody showed no abnormal cross reactivity.

## Results

### Monomeric C-Reactive Protein Staining Profiles

**Figure 1:**
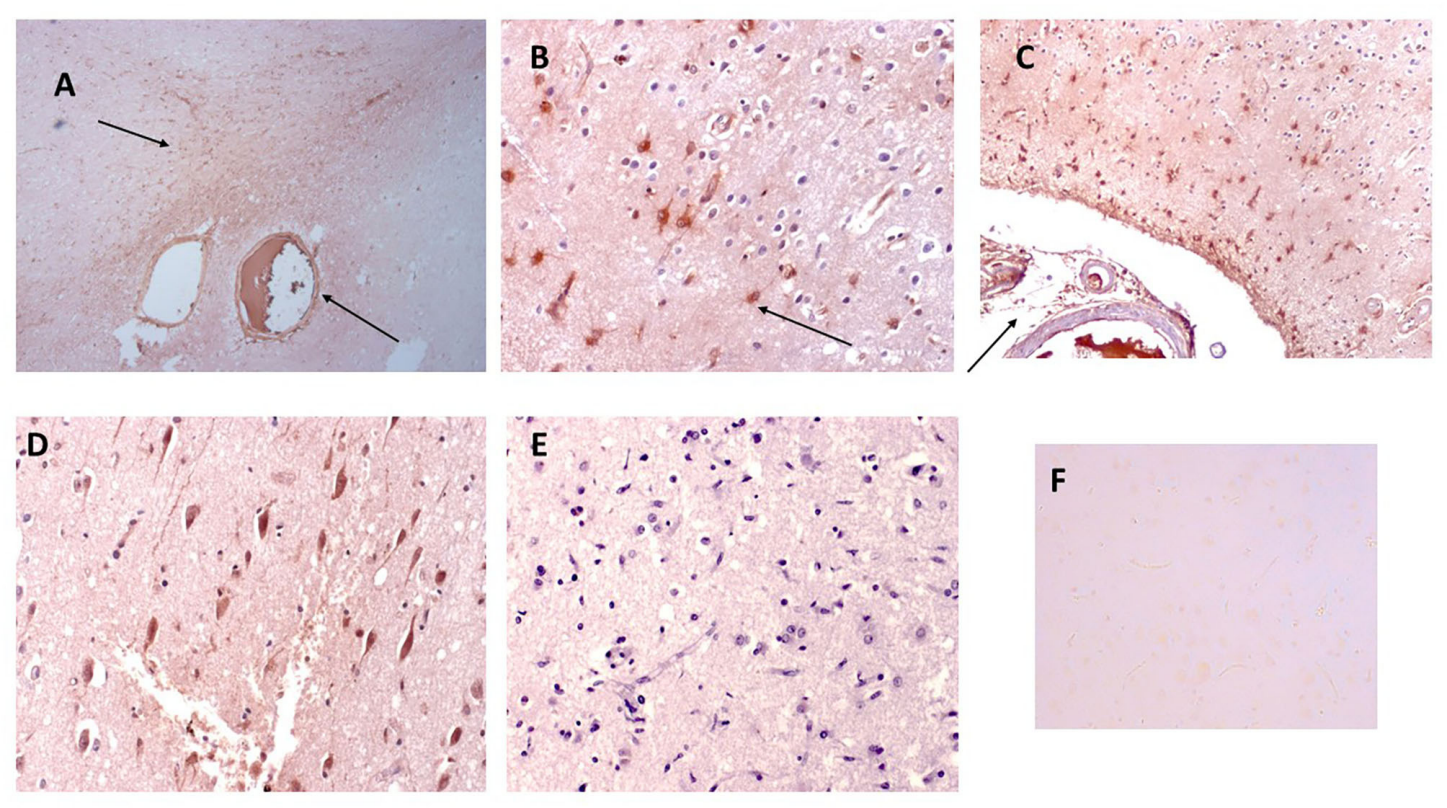
Shown here sample-883-BG; **(A)** cortical vessels covered with mCRP and spreading of mCRP positive staining into the local parenchyma (arrows x100). **(B)** Shows mCRP-positive cells with morphology of close by to the same region (arrows x200). **(C)** Shows medium sized blood vessels in the cortex saturated with mCRP and cortical infiltration becoming weaker as it moves further away from the vascular source (x100). **(D)** Shows a region of the cortex in sample 850-F where clusters of abnormal looking neurons are strongly stained with mCRP (x200), whilst **(E)** shows an area of normal-looking gray matter cortex that is devoid of mCRP staining (x200; hematoxylin counterstained). **(F)** Shows a negative control section where the primary antibody was replaced with PBS). mCRP staining is developed as DAB brown.

Of the 13 patient samples examined, 10 showed significant macroscopically visible and specifically mCRP positive regions that were associated with vascular structures, cortical vessels, and other regions of neurodegeneration and abnormal looking brain tissue in confirmed cases of AD. The major highlights were as follows:

Strong mCRP staining was seen around abnormal looking tissue with vascular disturbance and histological evidence of a strong local inflammatory response with many macrophages/glia stained positively for mCRP (shown here sample-883-BG; Figures 1A,B, respectively, arrows).

In Figure 1C, local cells to a large vessel leaded with mCRP also have notable mCRP positive staining that tapers out as it gets further away from the vascular source (x200; arrow)—In other areas mCRP was found loaded into intact vessels without obvious leakage (data not shown).

In distant cortical normal looking tissue regions, there was no evidence of mCRP staining in vessels, parenchyma or other cells (Figure 1E; x200 with hematoxylin counter staining).

Figure 1D (sample 850-F) shows considerable mCRP staining in cortical gray matter with degenerative appearance and clusters of other mCRP-positive neurons cells again suggesting leakage and local systemic transfer.

Similarly, in 893-F (no images supplied), there was a similar notable mCRP staining around abnormal looking or leaky blood vessels and evidence of positively stained cells with the morphological appearance of macrophages and microglia in small regions of abnormal looking tissue. These features were present in all the AD samples examined. Figure 1F (x200) shows an IgG control where the primary antibody development was replaced with PBS.

### Monomeric-CRP in the Hypothalamus

**Figure 2:**
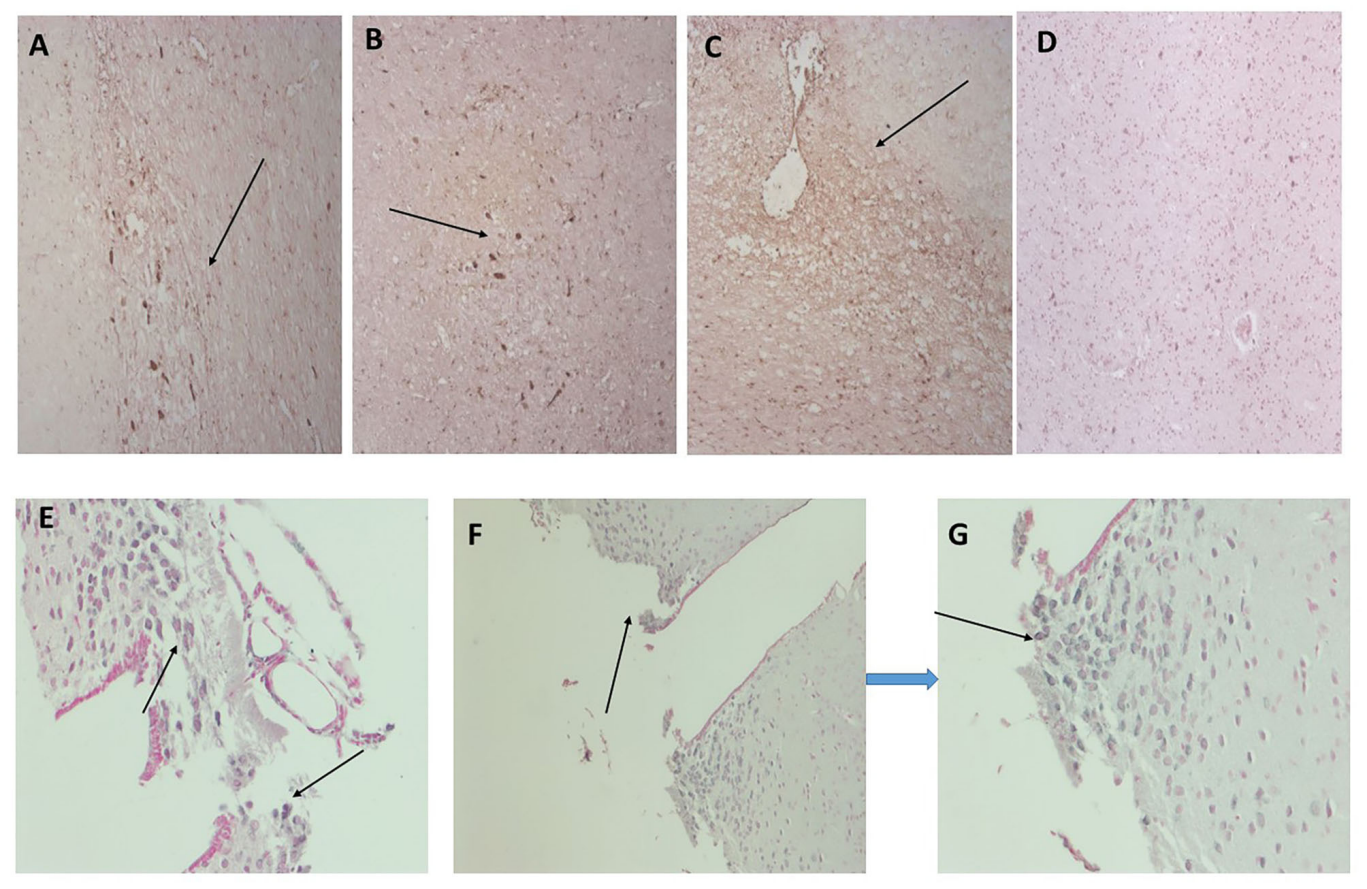
**(A–C)** Three images (x100) showing positive and cellular as well as ECM hypothalamic staining for mCRP in sample 839-HYPO (arrows). The parenchyma looks abnormal with tissue vacuolization, abnormal cell patterns compared with control normal looking tissue and loss of nucleated cells. **(D)** Shows normal looking hypothalamus tissue stained with hematoxylin (x100). **(E–G)** Show mCRP-antibody stained sections of mouse hippocampus 1 month after stereotactic injection of mCRP into the CA1 hippocampal region. The arrows point to mCRP-positively peri-nuclear stained neurons in this region not found in normal murine hypothalamus [see reference (13) for control stained sections et al.]. (**E** x200, **F** x100, and **G** x200; DAB blue-black development and fast red counterstain).

In Figure 2, we show surprising regions of mCRP-positive staining within the hypothalamus region of sample 839-HYPO (Figures 2A–C; x100; arrows). The cell staining is not defined here but the tissue looks strikingly abnormal compared with normal looking hypothalamus in which there was no evidence of mCRP staining (Figure 2D). We have previously shown also in mCRP-hippocampal injected mice, that somehow, mCRP becomes visible in the neuronal cytoplasm of cells in the hypothalamus and to demonstrate this point, have included Figure 2E from the article Slevin et al. (13) as well as additional photomicrographs from the same experiments that show clear neuronal mCRP-positivity from an identically treated mouse (Figure 2F; x200 and Figure 2G; x100 and 200, respectively, arrows).

### Co-localization With Neurodegenerative Markers

**Figure 3:**
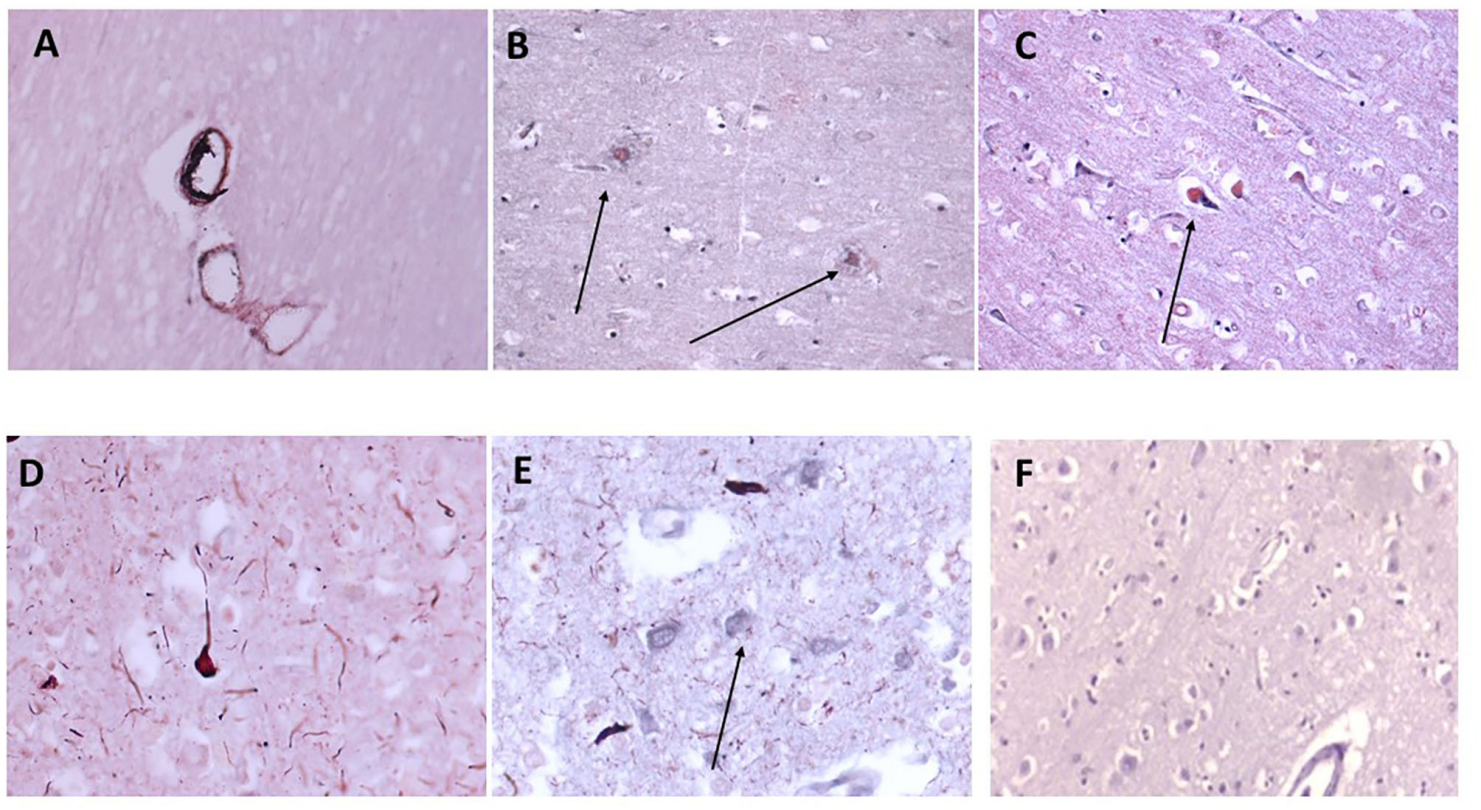
Shows an example of neurodegenerative features of this cohort of samples with double IHC staining in 697-PAR showing medium sized cortical vessels staining positively with anti-monomeric C-reactive protein antibody and B-amyloid (**A–C**, microvessels, plaques and neurons, respectively, x100; arrows) and co-localizing with p-Tau in neurons/fibrils (**D,E**, x200; arrows). **(F)** Shows a cortical region unaffected (no evidence of neurodegeneration) with relatively normal parenchymal and cellular architecture and no mCRP staining (x200) (mCRP staining developed with DAB blue-black and β-amyloid/p-Tau in nova red; arrows).

We have previously examined this cohort of samples for neurodegenerative protein expression (18). Firstly, we again confirmed co-localization of mCRP with β-amyloid in cortical microvessels, plaques, and neurons, respectively (Figures 3A–C; x100); mCRP blue-black/β-amyloid in nova red; 691-F as the provided example). Figures 3D,E show a similar co-localization in the same sample of mCRP with p-Tau, in neurons, neuritic plaques/fibrils and a higher power image of co-localization of both in a cortical neuron, respectively. Normal looking region of cortex with no visible mCRP staining (Figure 3F). Other cases showed similar patterns of staining (data not included).

### Mcrp Link to Neuroinflammatory Tissue Regions

In order to identify if the mCRP staining and mCRP protein presence was associated with the neuroinflammatory process, we carried out double labeling of the sections from several individuals to look for the concomitant increased localized expression of CD68 (macrophage/glia marker) and IL-1-beta (major inflammatory cytokine) and NF-κB (major inflammatory signaling transcription factor).

#### CD68

**Figure 4:**
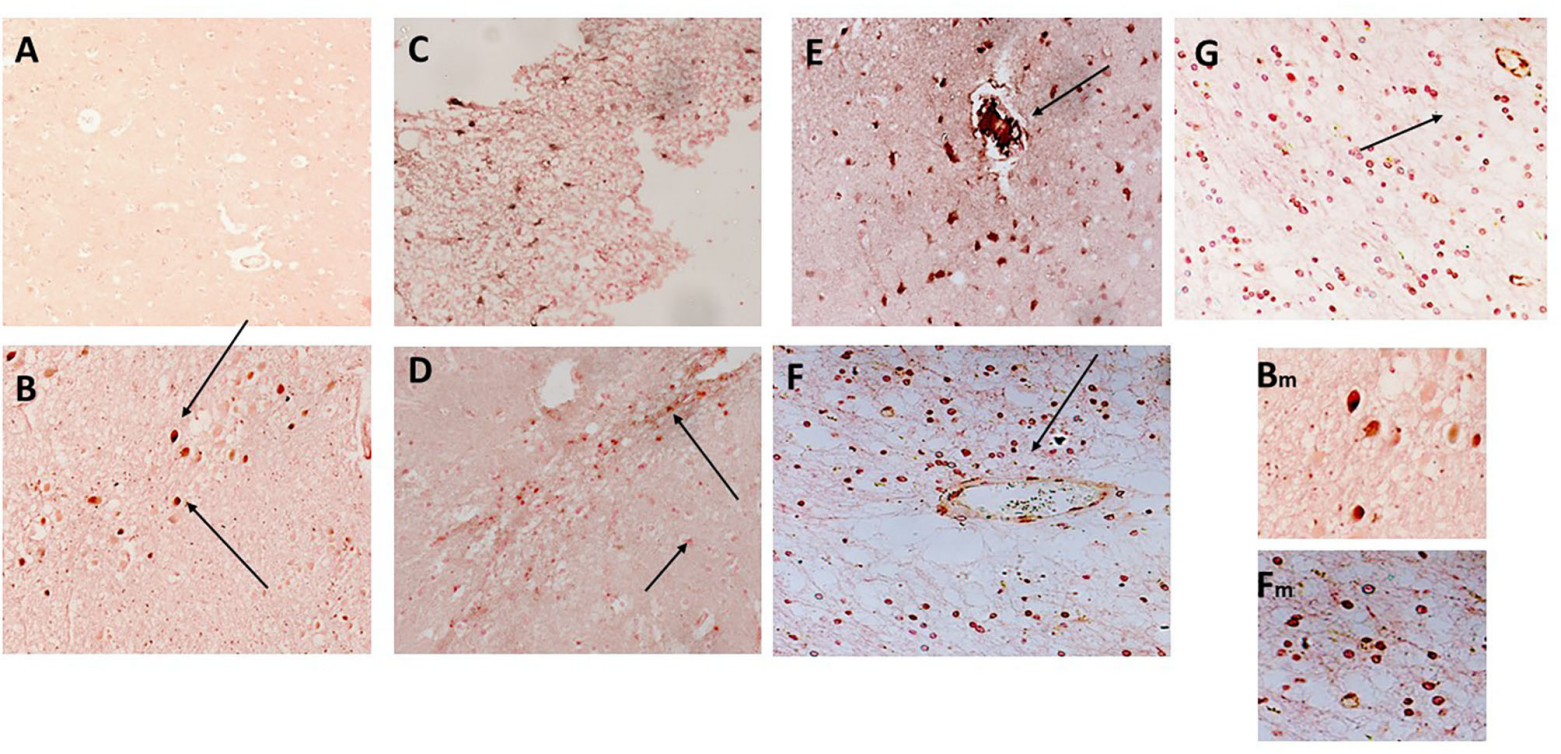
**(A)** Shows a normal portion of sample 798-PAR, cortical region where there was no mCRP nor CD68 staining (x100). However, in a separate region of tissue, **(B–D)** show strong staining of both mCRP and CD68 in this area that has evidence of either infarction nor other neurodegenerative damage or inflammation. There was strong co-localization of CD68 (nova red) and mCRP (DAB brown/black) in cells with the morphological appearance of glia/microglia (not proven with direct IHC) and associated microvessels (arrows; x200). Similarly, in 963-PAR: a region surrounding a damaged microvessel also stained strongly for CD68 and mCRP (**E–G**; x200), and small immune cells can be seen probably infiltrated into the region that show a mixture of mCRP and CD68 staining. Magnified exerts from **(B,F)** are shown at x300 to clearly show the bi-color staining pattern.

CD68 is a macrophage/microglia specific marker that has been shown to be increased in inflammatory regions of the brain during development of AD and other neurodegenerative conditions (19). In samples 798-PAR, there was no mCRP nor CD68 staining in the control contralateral hemisphere (Figure 4A; x100). However, a region of tissue with evidence of infarction/damage showed strong co-localized staining of CD68 (red) and mCRP (brown) in glia/microglia and associated microvessels (Figures 4B–D; arrows; x200). Similarly, in 963-PAR: a region surrounding a damaged microvessel also stained strongly for CD68 and mCRP (Figures 4E–G; x200).

#### IL-1β

**Figure 5:**
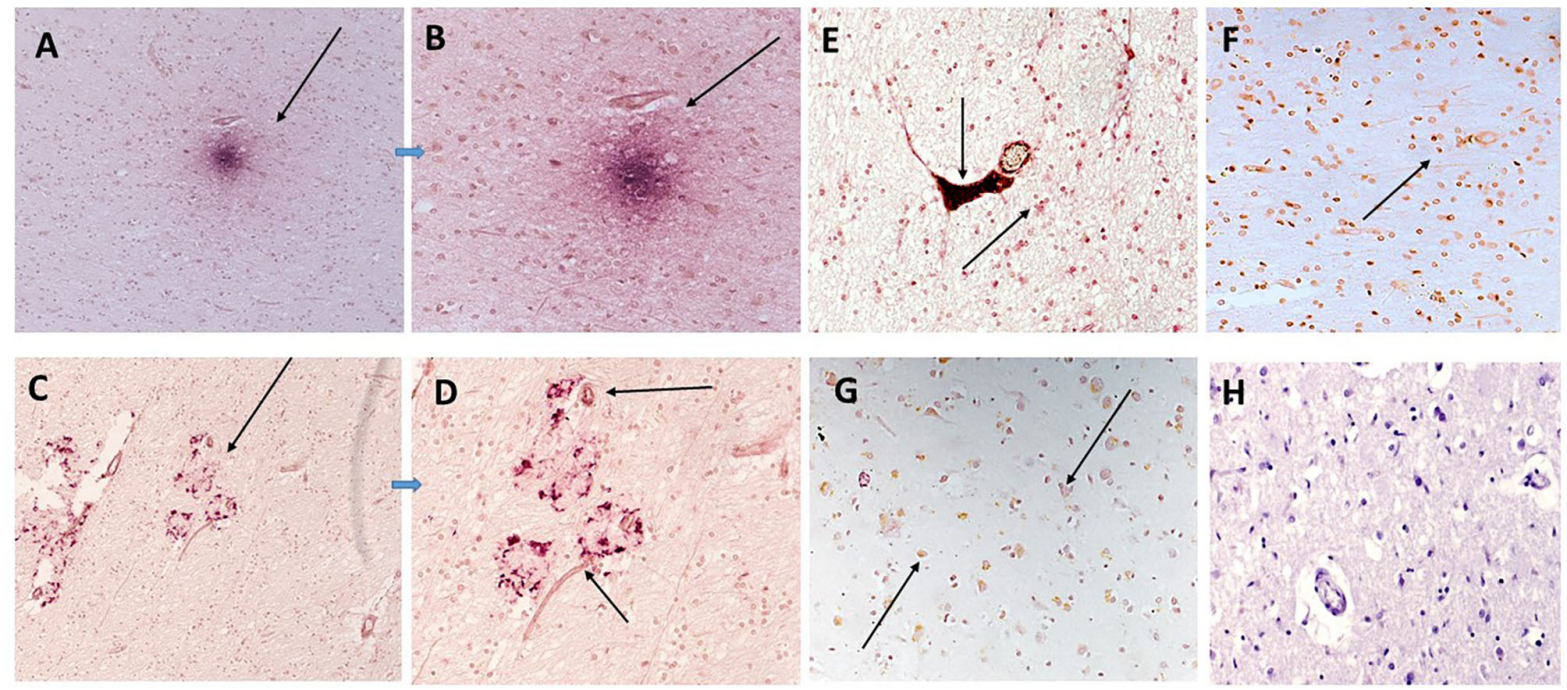
In sample 839-F **(A,B)** show co-localization of mCRP (DAB brown) with IL-1β (nova red) in an inflammatory cortical region with plaque-like structures (**A,B**; x100 and x200, respectively). Similarly **(C,D)** show strong staining of IL-1β in other plaque-like features (arrows) with microvessels also staining positive for mCRP (DAB brown). **(E)** Sample 697-F shows very strong mCRP staining in a region of white matter, here the arrows show a microvessels packed with mCRP (DAB brown) next to a smaller vessel also positively stained and surrounded by Il-1β/mCRP-positive immune like cells (x200, arrows). **(F,G)** Show a similar area of white matter and gray matter (respectively) with interspersed co-localized staining of both proteins in abnormal looking tissue regions (x100 arrows). **(H)** (x200) shows a normal looking region from patient sample 697F showing a lack of staining for either mCRP or IL-1β.

IL-1β staining was carried out for direct evidence of an on-going acute inflammatory reaction was evidenced by strong staining within plaque like structures (red) in patient sample 839-F, with co-localization of mCRP (brown) in surrounding vessels and other cells (Figures 5A,B; x100 and x200, respectively). Similarly, Figures 5C,D show strong staining of IL-1β in plaque-like features (red) with some regions and microvessels also staining positive for mCRP (arrows in Figure 5D; x200). Abnormally appearing white matter in patient sample 697-F showed areas with microvessels packed with mCRP (brown) surrounded by many Il-1β-positive smaller immune like cells (Figure 5E; x200; arrows). In Figures 5F,G, mCRP-positive cells (brown) are interspersed in the white matter with IL-1β ones (Figure 5F; x100; arrows) and in the gray matter with neurons (Figure 5G; x200; arrows). Figure 5H (x200; hematoxylin counter stain) shows a normal looking region from patient sample 697F with a lack of staining for either mCRP or IL-1β.

#### NFκB

**Figure 6:**
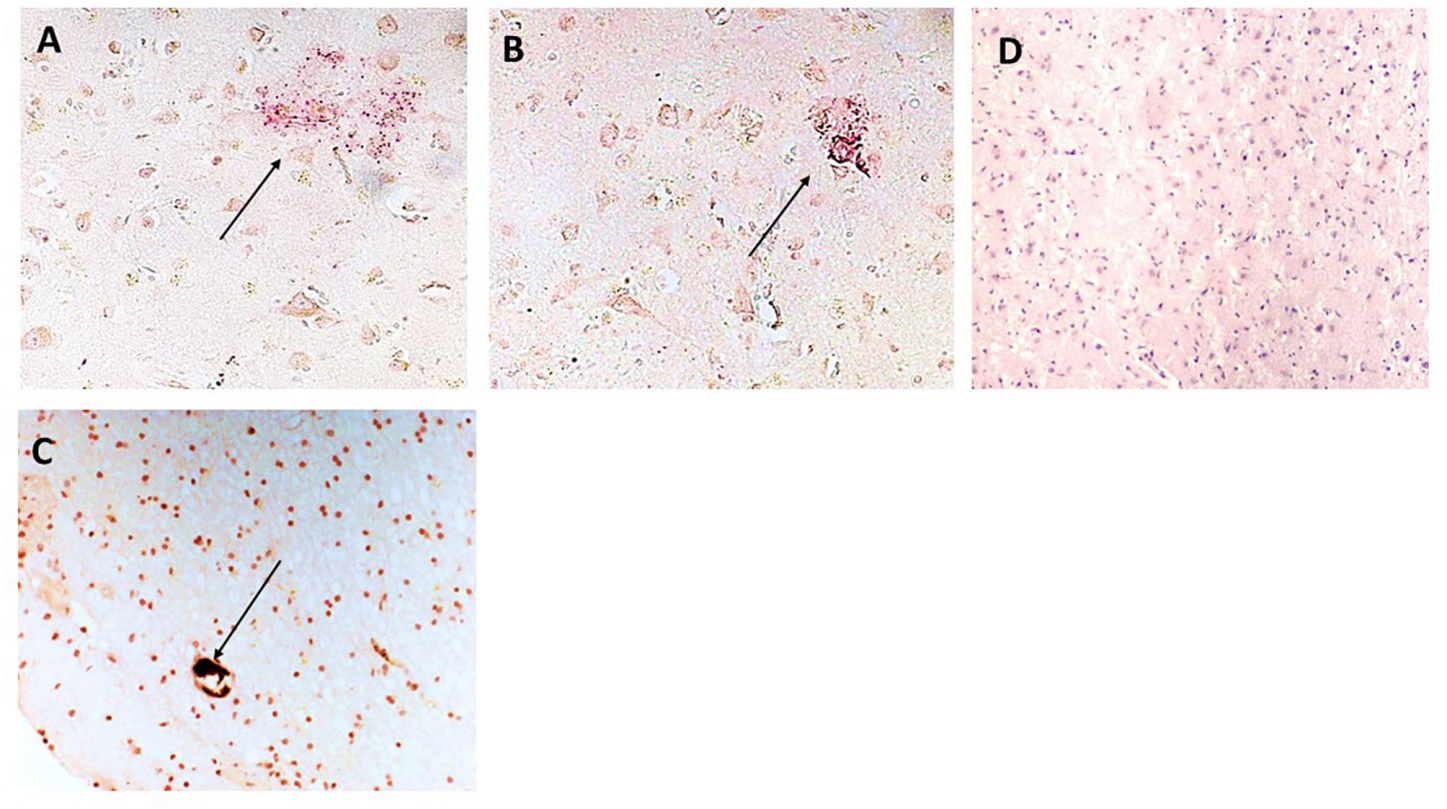
**(A,B)** Show sample 697-P; cortical region with plaque-like material staining positive for NFκB (arrows x200) and surrounded by mCRP-positive neurons and other cells (DAB brown). **(C)** Shows mCRP engulfed within a microvessel (DAB brown/black) surrounded by smaller immune/microglia (morphological appearance not directly proven with IHC) positive for both mCRP and phospho NFκB (x200; arrow). **(D)** Is a normal looking region from the same individual negative for staining in both mCRP and phospho-NFκB (x200).

NFκB staining was used to understand if signaling pathways associated with inflammatory activation were modified in regions of mCRP positivity. We showed that phospho-NFκB (red) was present in plaque-like structures that were surrounded by mCRP (brown)-positive neurons in the cortex (patient sample 697-P; Figures 6A,B; x200). Figure 6C shows mCRP engulfed within a microvessel (brown/black) surrounded by smaller immune/microglia positive for both mCRP and phosphor NFκB (697-P, x200). Figure 6D shows a contralateral normal looking hemisphere negative for staining in both mCRP and phospho-NFκB (x200; hematoxylin counterstain).

**Figure 7:**
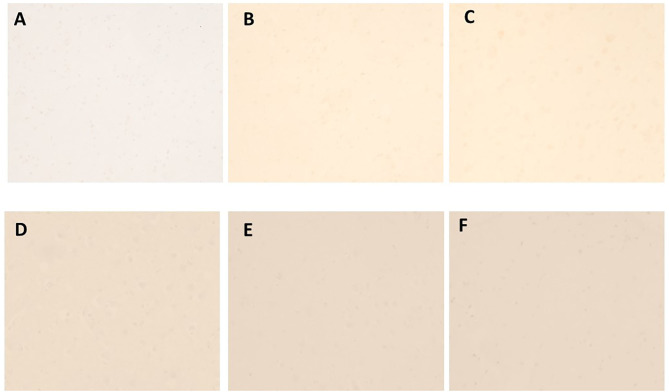
Shows negative control sections (x200; without counterstain) where the primary antibody was replaced with PBS; for serial section samples derived from 839-Hypo (**A)**, 691-F **(B)**, 798-PAR **(C)**, 963-PAR **(D)**, 839-F **(E)**, 697-F **(F)**, and 697-P **(G)**.

Figure 7 shows negative control sections (x200; without counterstain) stained using secondary antibody with replacement of the primary antibody; for serial section samples derived from 839-Hypo (A), 691-F (B), 798-PAR (C), 963-PAR (D), 839-F (E), 697-F (F), and 697-P (G).

### Correlation of Staining Patterns

Strongest staining of mCRP was found in regions of previous infarction and these were almost always associated with inflammatory infiltrations, glia and macrophages staining positive also for mCRP in the vicinity of “leaky” blood vessels seemingly covered or surrounded by mCRP. Further insight was gained by a demonstration that in these same regions signal transduction activation was seen by the presence of increased staining of NfKB. In normal looking regions of the cortex, there was no mCRPP as shown in our controls but surprisingly, and following on from our finding from the previously hippocampal-injected mice, we found mCRP in cells of the hypothalamus even where there was no other sign of internal tissue dis-organization or causative incident-this should be investigated in more detail.

## Discussion

This study is the first to demonstrate a strong link between mCRP deposition within the brain of AD patients and local neuro-inflammation associated with vascular leakage in abnormal regions of the brain showing conventional hall marks of AD. Here, we have specifically identified post-mortem cases from the Bristol brain bank that were diagnosed with AD or VaD and that subsequently we showed by histology to have also evidence of infarcted lesions. In this way we could assess the localization of mCRP in relation to neuroinflammation and cell damage.

The mCRP staining was seen primarily with regions of neuro-inflammation with cells of the morphological appearance of macrophages, other immune cells and also glia staining positive in the vicinity of both small and also larger penetrating vessels. We previously showed an image of what appears to be mCRP being released from the site of a larger vessel into the local parenchyma where it subsequently created a local mCRP-positive micro-environment within the parenchyma (20). This is an important observation as here and previously, we showed what appears to be a transmission of mCRP *via* the vasculature from the hippocampus to distant regions of the hypothalamus in addition to other regions of the cortex suggesting it can be carried from the original site of vascular penetration, possibly aiding or even instigating neuroinflammation across the brain. The extensive volume of mCRP, sometimes appearing to occlude the lumens of vessels, suggests an inability to remove this isoform with potential toxic build up and negative consequences for progression of disease.

Monomeric C-reactive protein was recently shown to directly exert a pro-inflammatory effect upon macrophages and glia, *via* induction of nitric oxide synthase (21) and TNF-α/IL-1β amongst other cytokines (22). Hence this could be a mechanism that could partially explain how the deposition of mCRP may support creation of a pro-inflammatory micro-environment perpetuating on going damage. Additionally, others have reported CRP/mCRP expression directly in AD-beta amyloid positive plaques suggesting a connection to neurovascular damage and neuritic plaque development (6). Here, we have shown that a strong local inflammatory response is seen surrounding the regions of mCRP deposition and the vasculature, with these regions showing strong chaotic morphology and concomitant expression of key inflammatory markers in addition to inflammatory infiltration.

In regard to the possible relationship to angiogenesis, aberrant angiogenesis associated with formation of none-patent or leaky microvessels as part of a regenerative or restorative effort within damaged brain tissue only serves to permeabilise the parenchyma thereby further increasing immune cell infiltration and so on and so forth (2, 22). It was recently shown that mCRP was able to disrupt the outer retinal blood brain barrier indicating a potential role in inflammatory macular degeneration (23), taken together, it appears that mCRP may have distinct effects both directly upon the vascular integrity and other pro-inflammatory stimulatory effects that combined could impact upon the normal function of the neurovascular unit. Whilst Strang et al. (12) and others have suggested the possibility of *de-novo* synthesis of mCRP within the brain, in this study we are suggesting that direct brain vascular damage and leakage from blood vessels is the major source of the mCRP, whilst movement throughout the brain seems also likely we cannot discount the possibility of glial or other cell synthesis of mCRP and this needs to be studied in more detail.

## Conclusion

We have shown mCRP-build-up within the microvasculature of brain tissue localized to neurodegenerative or past-infarcted regions. We have shown local release of mCRP into brain parenchymal tissue and a concomitant activation of regional and focal blood vessels, and strong co-staining with markers of inflammation and inflammatory infiltrating cells. In distant regions of the hypothalamus, mCRP also became visible and the consequences of this nor the mechanism at which it arrives there are still not understood. This study provides evidence in support of an important role for mCRP in vascular damage associated inflammation relating to later neurodegenerative consequences. Indirect effects on hypothalamic inflammation could have negative consequences associated with metabolic disease and cardiovascular consequences that should be examined in more detail. A limitation of this study is in the relatively small cohort size and in addition, the purely “snap-shot” indications that can be derived from this IHC approach do not categorically implicate a direct relationship between the mCRP presence and increased levels of inflammation or neurodegeneration seen here.

## Data Availability Statement

The original contributions presented in the study are included in the article/supplementary material, further inquiries can be directed to the corresponding authors.

## Ethics Statement

The studies involving human participants were reviewed and approved by further tissue samples (UK-Bristol Brain Bank, with ethical approval through application to https://mrc.ukri.org/research/facilities-and-resources-for-researchers/brain-banks/, and following their guidelines at https://mrc.ukri.org/publications/browse/human-tissue-and-biological-samples-for-use-in-research/; local approval was gained for the use of the tissue within the Manchester Metropolitan University LREC committee). The patients/participants provided their written informed consent to participate in this study. The animal study was reviewed and approved by all experiments were performed in accordance with ARRIVE Guidelines for the Care and Use of Laboratory Animals, and the Spanish guidelines/legislation concerning the protection of animals used for experimental and other scientific purposes and the European Commission Council Directive 86/609/EEC on this subject. All experimental protocols were approved by the above authority.

## Author Contributions

MS, NH, and RA-B co-ordinated the work and drafted the manuscript. DL, GF, SP, and EG-L conducted the IHC and histology. CS co-ordinated the animal work. SA, BA, AA-h, and FA analyzed the data and helped draft the manuscript. All authors contributed to the article and approved the submitted version.

## Conflict of Interest

The authors declare that the research was conducted in the absence of any commercial or financial relationships that could be construed as a potential conflict of interest.
